# Transcriptional and Phenotypic Characterization of Novel Spx-Regulated Genes in *Streptococcus mutans*

**DOI:** 10.1371/journal.pone.0124969

**Published:** 2015-04-23

**Authors:** Lívia C. C. Galvão, James H. Miller, Jessica K. Kajfasz, Kathy Scott-Anne, Irlan A. Freires, Gilson C. N. Franco, Jacqueline Abranches, Pedro L. Rosalen, José A. Lemos

**Affiliations:** 1 Center for Oral Biology, University of Rochester Medical Center, Rochester, New York, United States of America; 2 Department of Microbiology and Immunology, University of Rochester Medical Center, Rochester, New York, United States of America; 3 Department of Physiological Sciences, Dentistry School of Piracicaba, State University of Campinas, Piracicaba, SP, Brazil; 4 Department of General Biology, Laboratory of Physiology and Pathophysiology, State University of Ponta Grossa, Ponta Grossa, PR, Brazil; University of Kansas Medical Center, UNITED STATES

## Abstract

In oral biofilms, two of the major environmental challenges encountered by the dental pathogen *Streptococcus mutans* are acid and oxidative stresses. Previously, we showed that the *S*. *mutans* transcriptional regulators SpxA1 and SpxA2 (formerly SpxA and SpxB, respectively) are involved in stress survival by activating the expression of classic oxidative stress genes such as *dpr*, *nox*, *sodA* and *tpx*. We reasoned that some of the uncharacterized genes under SpxA1/A2 control are potentially involved in oxidative stress management. Therefore, the goal of this study was to use Spx-regulated genes as a tool to identify novel oxidative stress genes in *S*. *mutans*. Quantitative real-time PCR was used to evaluate the responses of ten Spx-regulated genes during H_2_O_2_ stress in the parent and Δspx strains. Transcription activation of the H_2_O_2_-induced genes (8 out of 10) was strongly dependent on SpxA1 and, to a lesser extent, SpxA2. *In vitro* transcription assays revealed that one or both Spx proteins directly regulate three of these genes. The gene encoding the FeoB ferrous permease was slightly repressed by H_2_O_2_ but constitutively induced in strains lacking SpxA1. Nine genes were selected for downstream mutational analysis but inactivation of *smu127*, encoding a subunit of the acetoin dehydrogenase was apparently lethal. *In vitro* and *in vivo* characterization of the viable mutants indicated that, in addition to the transcriptional activation of reducing and antioxidant pathways, Spx performs an important role in iron homeostasis by regulating the intracellular availability of free iron. In particular, inactivation of the genes encoding the Fe-S biogenesis SUF system and the previously characterized iron-binding protein Dpr resulted in impaired growth under different oxidative stress conditions, increased sensitivity to iron and lower infectivity in rats. These results serve as an entryway into the characterization of novel genes and pathways that allow *S*. *mutans* to cope with oxidative stress.

## Introduction

Dental caries remains one of the most prevalent infectious diseases affecting billions of people worldwide [1]. Caries results from an ecological imbalance of the oral flora caused by interactions of specific bacteria (e.g. *Streptococcus mutans*) with salivary proteins and dietary carbohydrates (e.g. sucrose). The cariogenic potential of microorganisms is largely based on three factors: (i) the ability to form biofilms on the tooth surface, (ii) the ability to produce weak acids mainly lactic acid, and (iii) the ability to rapidly adapt to environmental stresses such as large and fluctuations in pH, oxygen tension and nutrient availability [2]. In addition to fulfilling these central requirements, *S*. *mutans* appears as one of the most dominant species during the early and intermediate stages of caries development, strengthening its association with dental caries initiation and progression [3,4].

Well-documented work from a number of laboratories has established that *S*. *mutans* is well-equipped to adapt to low pH values by activation of a robust physiological response to acidification that includes, among other responses, upregulation of the membrane-associated F-ATPase, induction of pathways that contribute to cytoplasm buffering and changes in membrane fatty acid composition [2]. In addition to acid tolerance, mounting evidence indicates that the ability of *S*. *mutans* to cope with the reactive oxygen species (ROS) generated by their own metabolism as well as by other oral species can also impact its pathogenic potential. In fact, it has been shown that, in dental plaque, there is an inverse correlation between the total numbers of *S*. *mutans* and of members of the mitis group (e.g. *S*. *sanguinis* and *S*. *gordonii*), known to produce large quantities of H_2_O_2_ from the metabolic reduction of oxygen. Specifically, members of the mitis group are often associated with oral health; a series of elegant *in vitro* studies showed that H_2_O_2_ produced by *S*. *gordonii* or *S*. *sanguinis* serves as a “chemical weapon” antagonizing the growth of *S*. *mutans* [5]. Likewise, *S*. *oligofermentans*, a recently identified oral streptococci isolated from plaque of caries-free patients, was shown to antagonize the growth of *S*. *mutans* through a lactate oxidase that generates H_2_O_2_ from lactic acid [6]. Finally, H_2_O_2_ present in certain oral hygiene and tooth bleaching products may represent another source of peroxide stress for oral bacteria [7].

As a facultative anaerobe that lacks catalase and a full electron transport chain, oxygen metabolism in *S*. *mutans* is thought to occur through two flavin-based enzymes, Nox and AhpF [8]. However, biochemical and physiological characterization of AhpF (an H_2_O_2_-forming NADH oxidase) and Nox (an H_2_O-forming NADH oxidase) indicates that the latter is the stronger of the two implicated in oxygen metabolism [9,10]. Loss of Nox leads to a decreased ability to metabolize oxygen and elevated expression of genes involved in ROS detoxification [9]. In conjunction with the AhpC peroxidase, AhpF was shown to be part of an alkyl hydroperoxide reductase system working as a flavoprotein dehydrogenase, which supports peroxide reduction by AhpC [8]. Other ROS scavenging and protective systems are also present in *S*. *mutans* including superoxide dismutase (SodA), thiol peroxidase (Tpx), the thioredoxin reductase system (TrxA/TrxB), glutathione synthase (GshAB), glutathione oxidoreductase (Gor), and the iron-binding peroxide resistance protein Dpr [11–14].

Spx is a global regulator ubiquitously found in low GC Gram-positive bacteria that is involved in stress survival, principally by serving as a transcriptional activator of genes involved in thiol homeostasis and detoxification [15]. Spx lacks a DNA-binding domain and its function depends on direct interactions with the RNA polymerase α-subunit, which can result in either positive or negative regulation [16,17]. There is now clear evidence that two or more Spx paralogs, with overlapping but also unique regulatory functions, contribute to adaptive stress responses of streptococcal species and, more recently, *Bacillus anthracis* [18–23].

Previously, we identified and characterized two Spx proteins (SpxA1 and SpxA2, previously named SpxA and SpxB, respectively) in *S*. *mutans* [18,19]. Inactivation of *spxA1* significantly impaired growth and survival under acid and oxidative stress conditions. Whereas stress tolerances were generally not impaired in the Δ*spxA2* strain, the stress sensitivities of the double Δ*spxA1*Δ*spxA2* strain was more pronounced than in the Δ*spxA1* strain [19]. Microarray profiling of the Δ*spxA1*, Δ*spxA2* and Δ*spxA1*Δ*spxA2* strains further indicated that SpxA1 plays a primary role in activating oxidative stress genes whereas SpxA2 appears to have a secondary role in the regulation of these same genes [19]. Notably, nearly every known gene of *S*. *mutans* with a proven role in oxygen metabolism or oxidative stress was found to be under Spx control. For example, transcription of *ahpC*, *ahpF*, *dpr*, *gor*, *nox*, *sodA*, *tpx* and *trxB* was downregulated in the Δ*spxA1* strain and, in most cases, further downregulated in the double mutant Δ*spxA1*Δ*spxA2* [19]. Despite the large number of genes identified in our microarray analysis, a relatively small number of genes followed this regulatory pattern, i.e. downregulated in both Δ*spxA1* and Δ*spxA1*Δ*spxA2* strains. We reasoned that some of the uncharacterized genes under SpxA1 control, and to a lesser extent SpxA2, might be involved in oxidative stress management. We performed transcriptional and phenotypic characterization of selected Spx-regulated genes previously identified in our microarray analysis to further investigate oxidative stress management under Spx regulation in *S*. *mutans*. Our results strongly suggest that, in addition to reducing and antioxidant pathways, Spx also controls metal ion homeostasis, thus serving as a starting point for the characterization of novel genes and pathways that allows for *S*. *mutans* to cope with oxidative stresses.

## Materials and Methods

### Bacterial strains and stress growth conditions

The bacterial strains used in this study are listed in Table 1. *S*. *mutans* UA159 and its derivatives were routinely grown in brain heart infusion (BHI) or trypticase soy agar (TSA) at 37°C in a 5% CO_2_ atmosphere or under anaerobic conditions (BBL Gaspack system, BD). Where appropriate, kanamycin (1 mg ml^-1^), erythromycin (10 μg ml^-1^) or spectinomycin (1 mg ml^-1^) was added to the growth medium. For mRNA quantifications, cultures were grown to OD_600_ = 0.4, at which point control samples were harvested by centrifugation, while experimental samples were exposed to 0.5 mM H_2_O_2_ for 5 and 15 min before harvest.

**Table 1:** Bacterial strains used in this study.

Strains	Relevant genotype	Source or reference
UA159	Wild type	Laboratory stock
Δ*spxA1*	*spxA1*:: Sp^R^	Kajfazs *et al*., 2010
Δ*smu143*	*smu143c*::Em^R^	This study
Δ*smu144*	*smu144c*:: Km^R^	This study
Δ*suf*	*smu248*::Km^R^	This study
Δ*dpr*	*smu540*::Em^R^	This study
Δ*feo*	*smu570*::Km^R^	This study
Δ*smu929*	*smu929c*::Em^R^	This study
Δ*smu1296*	*smu1296*::Km^R^	This study
Δ*smu1645*	*smu1645*::Km^R^	This study
Δ*spxA1*Δ*smu143*	*spxA1*:: Sp^R^, *smu143c*::Em^R^	This study
Δ*spxA1*Δ*smu144*	*spxA1*:: Sp^R^, *smu144c*::Km^R^	This study
Δ*spxA1*Δ*suf*	*spxA1*:: Sp^R^, *smu248*::Km^R^	This study
Δ*spxA1*Δ*smu929*	*spxA1*:: Sp^R^, *smu929c*::Em^R^	This study
Δ*spxA1*Δ*smu1296*	*spxA1*:: Sp^R^, *smu1296*::Km^R^	This study
Δ*spxA1*Δ*smu1645*	*spxA1*:: Sp^R^, *smu1645*::Km^R^	This study

Harvested pellets were stored at -80°C until use. To generate growth curves, strains were grown overnight under anaerobic conditions and diluted 20-fold in pH 7.0-buffered BHI containing H_2_O_2_ (0.2 mM), methyl viologen (MV, 5 mM), diamide (0.5 mM). Cultures were incubated at 37°C in a 5% CO_2_ atmosphere and the OD_600_ recorded at selected intervals. To test the toxicity of iron to *S*. *mutans* strains, bacterial cultures grown to OD_600_ ~ 0.3 were diluted 1:100 in fresh BHI supplemented with 5 mM FeSO_4_. After 18h of incubation at 37° in 5% CO_2_, a 100 μl aliquot of each culture was serially diluted and plated onto BHI agar for CFU counting. To test the sensitivity of *S*. *mutans* UA159 and its derivatives to streptonigrin in disc diffusion assays, a uniform layer of exponentially-grown cells was spread onto BHI agar plates using a sterile swab, and filter papers (Whatman paper, 1 mm in diameter) saturated with 20 μl of a solution of 0.125 μg μl^-1^ of streptonigrin were placed onto the agar. All plates were incubated at 37°C in a 5% CO_2_ for 48 h after which the diameter of the inhibition halo was measured.

### Construction of mutant strains

Standard DNA manipulation techniques were used as previously described (Sambrook *et al*., 1989). The *S*. *mutans* strains lacking the various genes selected for this study (*smu143c smu144c*, *smu248*, *smu540*, *smu570*, *smu929*, *smu1296* and *smu1497*) were constructed using a PCR ligation mutagenesis approach (Lau et al., 2002). Briefly, PCR fragments flanking each target gene were obtained and ligated to non-polar kanamycin (NP-Km^R^), polar kanamycin (Ω-Km^R^), or polar erythromycin (Em^R^) cassettes, and the ligation mix was used to transform *S*. *mutans* UA159. Double mutants were obtained by amplifying the mutated *spxA1* region in the previously constructed *ΔspxA1* strain (spectinomycin-resistant, Spc^R^) and using this PCR product to transform the different single mutants. Mutant strains were isolated on BHI plates supplemented with the appropriate antibiotic. The deletions were confirmed as correct by PCR sequencing of the insertion site and flanking region. Primers used to generate mutants are listed in S1 Table.

### Real-time quantitative RT-PCR

Total mRNA was obtained using our standard protocols [24]. Briefly, RNA was isolated from homogenized *S*. *mutans* cells by repeated hot acid-phenol:chloroform extractions, and the nucleic acid precipitated with 1 volume of ice-cold isopropanol and one-tenth volume of 3 M sodium acetate (pH 5) at 4°C overnight. RNA pellets were resuspended in 80 μl nuclease-free H_2_O, and treated with DNase I (Ambion) at 37°C for 30 minutes. The RNA was purified again using the RNeasy mini kit (Qiagen), including a second on-column DNase treatment that was performed as recommended by the supplier. RNA concentrations were determined in triplicate using a Nanovue Spectrophotometer (GE Healthcare) and run on an agarose gel to verify RNA integrity. For qRT-PCR, cDNA templates were created from 0.4 μg of RNA using the SuperScript first-strand synthesis system (Invitrogen). Reactions were carried out on a StepOnePlus real-time PCR system (Life Technologies) according to protocols described elsewhere [24]. Gene-specific primers (S2 Table) were designed using Beacon Designer 2.0 (Premier Biosoft International) to amplify region of each gene 85 to 200 bp in length.

### Protein Purification

The *S*. *mutans* SpxA1 and SpxA2 were expressed in *E*. *coli* as recombinant His-tagged fusion proteins using the pET-16B expression vector (EMD Millipore) as described elsewhere (Kajfasz *et al*, submitted). Purification of the recombinant proteins was performed by column chromatography using Ni-NTA resin (Qiagen) following the manufacturer’s instructions. A crude extract of *S*. *mutans* RNAP was obtained based upon the methods described by Seepersaud *et al* for the extraction of RNAP from *Streptococcus agalactiae*, which utilizes the heparin binding properties to enrich for RNAP [25]. Briefly, *S*. *mutans* cells were grown in BHI broth to OD_600_ ~ 0.5. Cells were harvested, resuspended in protoplast preparation buffer (0.3 M potassium phosphate buffer, pH 7.0; 40% sucrose; 0.5 U μl^-1^ mutanolysin) and incubated at 37°C for 90 min. Protoplasts were harvested, and the pellets resuspended in lysis buffer (50 mM Tris HCl, 10 mM MgCl_2_, 0.1 M DTT, 0.1 mM EDTA, 10% glycerol, 1 mM PMSF, pH 8.0), followed by 3 rounds of 15 seconds of sonication, intersperced by 1 minute intervals on ice. Following centrifugation, supernatants were applied to Affi-Gel heparin resin (Bio-Rad) and eluted with a gradient of a NaCl gradient of 0.1 to 1 M. The elutions were run on 10% SDS-PAGE and the fractions containing a visible β subunit (134 kDa) were pooled, and the salt concentration adjusted to 0.1 M NaCl by buffer exchange. The protein was then applied to Macro-Prep High-Q ion exchange resin (Bio-Rad) and eluted with a gradient of 0.1–0.8 M NaCl. The desired fractions were again pooled and dialyzed (10 mM Tris HCl, 10 mM MgCl_2_, 100 mM NaCl, 0.1 mM EDTA, 50% glycerol, pH 8.0). Protein concentration was determined using the bicinchoninic acid assay (Pierce).

### *In vitro* transcription assay

A linear DNA template for each gene of interest was generated by PCR using primers designed to amplify from the promoter region to 70 to 200 base pairs of the coding region (S3 Table). Following amplification, the DNA fragments were purified with a QIAQuick PCR Purification Kit (Qiagen). The *in-vitro* transcription (IVT) reactions were performed as described elsewhere [26]. Briefly, 10 nM of each individual purified promoter template, 25 nM *S*. *mutans* RNAP, and 25 nM *B*. *subtilis* σ^A^ (a gift from Peter Zuber, Oregon Health Science Center) were incubated with or without purified 75 nM ofSpxA1 or SpxA2 in reaction buffer (10 mM Tris-HCl, 50 mM NaCl, 5 mM MgCl_2_; pH 8.0) containing bovine serum albumin (BSA, 50 μg ml^-1^) to a final volume of 20 μl for 10 min at 37°C. A nucleotide mixture (200 mM ATP, GTP, and CTP, 10 mM UTP, and 5 μCi [α-^32^P] UTP) was added and the incubation proceeded for an additional 3 to 5 minutes. Stop solution (1 M ammonium acetate, 0.1 mg ml^-1^ yeast RNA, 0.03 M EDTA) was added and the mixture was precipitated with ethanol at 4°C for 3 hours. The nucleotide pellet was resuspended in formamide dye (0.3% xylene cyanol, 0.3% bromophenol blue, 12 mM EDTA dissolved in formamide). The samples were heated at 90°C for 2 min, then placed on ice before applying to an 8% polyacrylamide urea gel. The transcripts were visualized with Bio-Rad Quantity One Fx software following overnight exposure to an Imaging Screen-K (Bio-Rad).

### Competition assay on solid media

The ability of *Streptococcus gordonii* to inhibit growth of *S*. *mutans* via H_2_O_2_ production was assessed as described previously by Kreth *et*. *al*. [5]. Briefly, 8 μl of an overnight culture of *S*. *gordonii* DL-1 was spotted on the center of a BHI agar plate incubated at 37°C for 16 h. The following day, 8 μl of *S*. *mutans* cultures were spotted near the *S*. *gordonii* spot and incubated for an additional 16 h before visualizing the ability of the different *S*. *mutans* strains to grow in proximity of *S*. *gordonii*. To ascertain that any growth inhibition was due to the production of H_2_O_2_ by *S*. *gordonii*, a control condition was included in which 8 μl of catalase (0.75 μg μl^-1^) was immediately spotted on top of the *S*. *gordonii* culture.

### Rat colonization

The ability of the mutant strains to colonize the teeth of rats was evaluated using an established model of dental caries [27]. Pathogen-free Wistar rat pups 19 days of age were purchased from CEMIB/ University of Campinas (credited by ICLAS—International Council for Laboratory Animal Science). Rats were first screened to ensure a lack of indigenous mutans streptococci as previously described [27], and infected separately for three consecutive days by means of cotton swab with actively growing *S*. *mutans* strains. Animals were fed a cariogenic diet (Diet-2000 containing 56% sucrose) and 5% sucrose water (wt/vol) *ad libitum* to enhance the infection by *S*. *mutans*. The experiment proceeded for 15 days, at the end of which the animals were killed by CO_2_ asphyxiation, and the lower jaws removed for microbiological assessment. The number of mutans streptococci recovered from the animals was expressed as CFU ml^-1^ of jaw sonicate. This study was reviewed and approved by the University of Campinas Committee on Animal Resources (CEUA Protocol #2639–1).

### Biofilm assay

Biofilm formation of the constructed *S*. *mutans* mutants was assessed by growing cells in wells of polystyrene microtiter plates using a semi-defined biofilm medium (BM) [28]. Strains grown in BHI medium to OD_600_ ~ 0.5 were diluted 1:100 in BM containing 1% sucrose and added to the wells of the microtiter plate. Plates were incubated at 37°C in a 5% CO_2_ atmosphere for 24 h. After incubation, plates were washed twice with water to remove planktonic and loosely bound bacteria, and adherent cells were stained with 0.1% crystal violet for 15 min. The bound dye was extracted with 33% acetic acid solution, and biofilm formation was then quantified by measuring the optical density at 575 nm. To exclude the possibility that differences in bound cells obesrved were due to variations in growth rates and growth yields, the biofilm data was normalized by the final OD_600_ growth.

### Statistical analysis

Student’s *t* tests were performed to assess the distributions of the real-time PCR quantifications. One-way analysis of variance (ANOVA) was performed to verify the significance of the growth curves, growth in FeSO_4_, streptonigrin disc inhibition and biofilm quantifications. The animal study data were subjected to ANOVA in the Tukey-Kramer Honest Standard Deviation (HSD) test for all pairs. In all cases, *P*-values ≤ 0.05 were considered significant.

## Results

### Transcription of several Spx-regulated genes is induced by H_2_O_2_ stress

**Fig 1:**
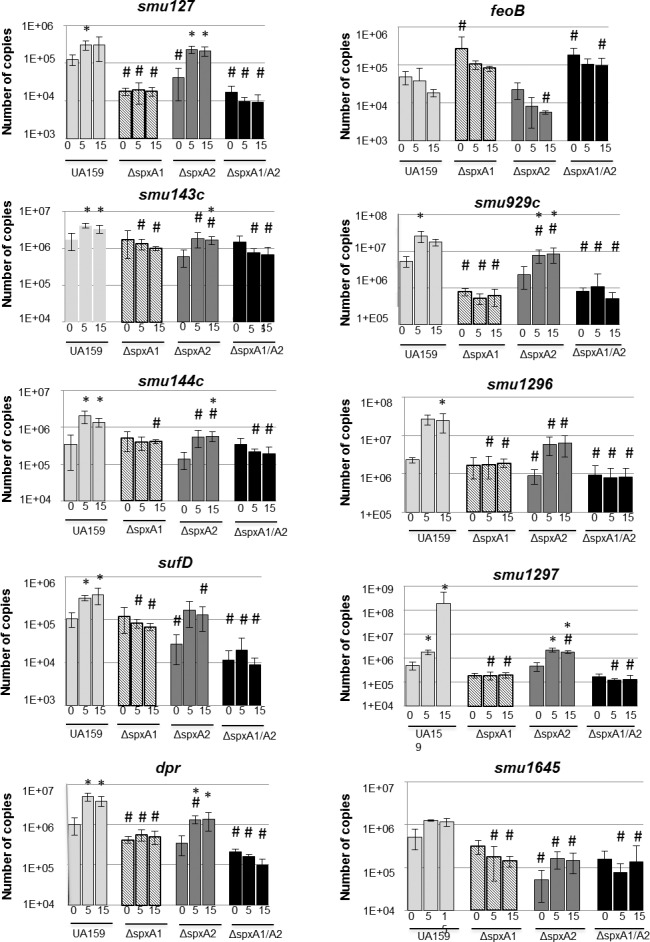
Transcriptional profile of Spx-regulated genes during H_2_O_2_ exposure. *S*. *mutans* UA159, Δ*spxA1*, Δ*spxA2*, or Δ*spxA1*Δ*spxA2* were grown in BHI to OD_**600**_ = 0.4. At that point, control cultures (Time 0) were harvested, while the remaining samples were exposed to 0.5 mM H_**2**_O_**2**_ for 5 and 15 minutes before harvest. Bars represent the relative copy number detected for each gene. (*) indicates a significant difference (*p* ≤ 0.05) from the same strain at Time 0; (^#^) indicates significant difference (*p* ≤ 0.05) from UA159 at the corresponding condition.

Nine genes selected for this study were previously identified in a microarray study as being positively regulated by the *S*. *mutans* SpxA1 protein [19]. In addition to the genes positively regulated by SpxA1, one gene under SpxA1 negative control, *smu570* predicted to encode the second gene (*feoB*) of the FeoABC iron transporter system [29], was also analyzed. The selected genes with their respective assigned functions and expression ratios from our previous microarray analysis are shown in Table 2. Recently, we showed that treatment with 5 mM H_2_O_2_ strongly induced the expression of known oxidative stress genes (e.g. *ahpC*, *gor*, *nox*, *sodA*, *tpx* and *trxB*), and that this regulation was strongly dependent on SpxA1 (Kajfasz et al., submitted). Here, we used quantitative real-time PCR (qPCR) to evaluate the transcriptional responses of the ten selected SpxA1-regulated genes (*smu127*, *smu143c*, *smu144c*, *smu248*, *smu540*, *smu570*, *mu929c*, *smu1296*, *smu1297* and *smu1645* to H_2_O_2_ stress (Fig 1). A heat map depicting the transcript fold change in relation to the parent UA159 under control growth conditions (T0) is also shown (S1 Fig). In the parent strain, transcription of *smu127*, *smu143c*, *smu144c*, *smu248* (*sufD*), *smu540* (*dpr*), *smu929*, *smu1296* and *smu1297* was significantly induced by H_2_O_2_ at both 5 and 15 min time points. Transcription of *smu1645* was also moderately induced by H_2_O_2_ but the differences were not statistically significant. Nevertheless, transcriptional levels of *smu1645* were significantly lower in all Δ*spx* stains confirming that this gene is also under Spx control. Among the H_2_O_2_-induced genes, *smu1296* and *smu1297* respectively, showed the most robust responses ranging from 10 to 100-fold induction after stress. Transcription of all H_2_O_2_-induced genes was strongly dependent on SpxA1 and, with the exception of *smu127*, SpxA2. In agreement with our previous microarrays, expression of *smu570* (*feoB*) was induced in the absence of SpxA1 but slightly repressed in Δ*spxA2*. Also within the expected, *feoB* transcription was repressed by H_2_O_2_ in the two SpxA1-positive strains (UA159 and Δ*spxA2*). Altogether, these results reveal that transcription of the genes investigated is controlled by both SpxA1 and SpxA2, induced by H_2_O_2_ in an SpxA1-dependent manner and, therefore, likely to participate in oxidative stress responses.

**Table 2:** List of selected genes under SpxA1/A2 control identified in microarray analysis.

Gene ID	Assigned function	Fold change in expression in Δ*spx* strains relative to UA159^a^		
		Δ*spxA1*	Δ*spxA2*	Δ*spxA1/A2*
*smu127*	acetoin dehydrogenase, *adhA*	0.206	ND^b^	0.131
*smu143c*	polypeptide deformylase	0.430	ND	0.425
*smu144c*	transcriptional regulator	0.255	ND	0.201
*smu247*	Fe-S assembly ATPase, *sufC*	0.578	ND	0.397
*smu540*	peroxide resistance protein, *dpr*	0.133	ND	0.198
*smu569*	ferrous ion transport, *feoA*	3.135	ND	2.253
*smu929c*	hypothetical protein	0.135	ND	0.108
*smu1296*	glutathione S-transferase	0.301	ND	ND
*smu1297*	pAp phosphatase	0.232	ND	0.325
*smu1645*	tellurite resistance protein, *tehB*	0.502	ND	0.245

^a^ [19]

^b^ ND, no difference.

### IVT assays confirm direct regulation of *smu144c*, *dpr* and *smu929c* by Spx

**Fig 2:**
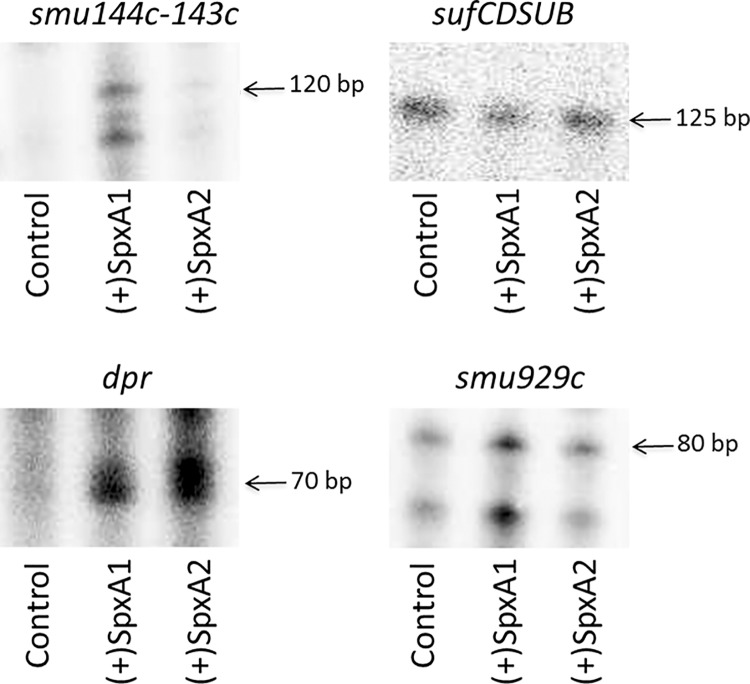
One or both Spx proteins specifically enhance transcription of *smu144c*, *dpr* and *smu929c* but not of the *suf* operon. *In-vitro* transcription reactions were performed by incubating the regulatory regions of stress genes with RNAP and nucleotides including [α-^32^P] UTP in the absence (control lane) or presence of 75 nM of purified SpxA1 or SpxA2 proteins at 37°C. Reactions were performed at 37°C. Radiolabeled RNA transcripts were precipitated, applied to 8% urea PAGE, and visualized by exposure to phosphorimager screen.

To confirm whether the regulatory effects exerted by the two Spx proteins were direct or indirect, we performed IVT reactions in the presence or absence of purified SpxA1 or SpxA2 proteins. Our results demonstrate that the addition of purified SpxA1 to the reactions enhanced transcription of *smu144c/143c*, *dpr* and *smu929c* (Fig 2). Addition of SpxA2 also enhanced *dpr* transcription. However, SpxA2, at equal or greater concentrations than SpxA1, had no apparent effects on *smu144c/143c* or *smu929c* transcript levels. In the case of the *sufA* operon, a transcript was seen in the absence of Spx protein, but the addition of SpxA1 or SpxA2 did not enhance transcription. Despite multiple attempts, we were unable to detect transcripts upstream *smu127*, *feoA*, *smu1296* and *smu1645* (data not shown). Given that transcripts for these genes have been detected by microarrays and qPCR, it is possible that these transcripts are not abundant and/or short-lived for detection via IVT assay. Another possible explanation is that co-factors or other regulatory proteins, not included in the *in vitro* system, may be required for efficient transcription of these genes.

### Growth characteristics of mutant strains

Upon confirmation that the selected genes were transcriptionally regulated by Spx and, in most cases, induced by H_2_O_2_ stress, we sought to determine the effect that deletion of each individual gene would have upon *S*. *mutans* ability growth under oxidative stress. The *smu1297* gene, encoding an enzyme with 3’-phosphoadenosine-5’-phosphate (pAp) activity, was previously identified in a transposon mutagenesis library to be involved in the superoxide stress response [30]. Because Smu1297 already has an assigned role in oxidative stress, the *smu1297* gene was not further investigated. Deletion mutations of eight of the genes described above were readily obtained but a viable *smu127* mutant strain was not. The *smu127* gene is predicted to encode the E1 component of the subunit α of the acetoin dehydrogenase (Adh) complex. The genetic organization of the eight mutated genes is depicted in (S2 Fig). The *smu143c* and *smu144c* genes, encoding, respectively, polypeptide deformylase and a putative transcriptional regulator, are separated by only 25 bp and co-transcribed (S3 Fig). Likewise, *smu1296* encoding a glutathione S-transferase (GST) homologue was shown to be co-transcribed with *smu1297* [30]. To avoid downstream polar effects, *smu144c* and *smu1296* were inactivated with a non-polar cassette that contains a promoterless *aphA3* Km^R^ gene without transcription termination sequences to allow transcription readthrough into downstream sequences [31]. Real-time PCR quantifications confirmed expression of the downstream *smu143c* and *smu1297* genes in the Δ*smu144* and Δ*smu1296* strains, respectively (S4 Fig). The Em^R^ marker was used to replace the *smu143c* and *smu929c* genes; *smu929c* is the last gene of a bicistronic operon (*smu930c*-*smu929c*). The *smu569-571* and *smu247-251* genes are arranged in operons with other genes involved in iron transport (FeoABC) and iron-sulfur (Fe-S) cluster assembly (SUF), respectively. To disrupt the FeoABC and Suf systems, the *feoA*/*feoB* genes (*smu569-570*) was replaced by the nonpolar Km^R^ cassette (Δ*feo*) whereas the first two genes in the *suf* operon (*sufC*/*sufD*) were individually inactivated using a polar Km^R^ cassette such that transcription of the downstream genes was interrupted (Δ*suf*). The *smu1645* gene, encoding a putative tellurite resistance protein, is transcribed in the opposite orientation of its surrounding genes and was also inactivated using the polar Km^R^ marker. Finally, we used the Em^R^ marker to inactivate the *smu540* gene, encoding the iron-binding peroxidase resistance Dpr protein. The importance of Dpr to *S*. *mutans* oxidative stress tolerance has been relatively well documented [11,32,33], and the Δ*dpr* strain was used as a benchmark in downstream studies.

**Fig 3:**
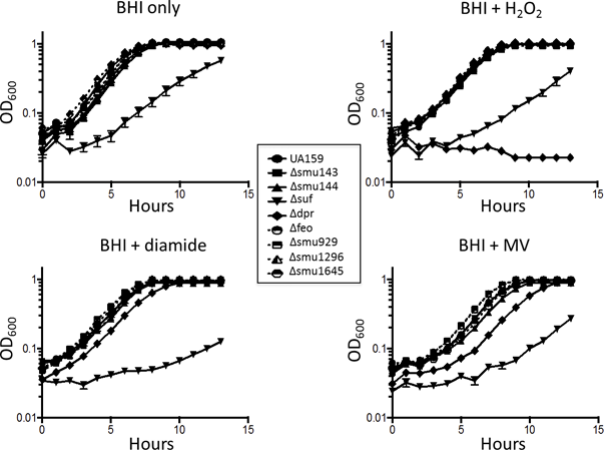
Growth curves of *S*. *mutans* UA159 and its derivatives under different environmental conditions. The curves shown are the means with standard deviations of the results from five independent cultures.

The growth characteristics of the mutant strains under different conditions are shown in Fig 3. Under standard non-stressful conditions, e.g. brain heart infusion (BHI) broth at 37°C in a 5% CO_2_ atmosphere, all strains, with the exception of the Δ*suf* strain with a doubling time of 2.56 ± 0.23 h, grew as well as the parent UA159 strain (doubling time 1.32 ± 0.07 h). Next, we evaluated the ability of the mutant strains to grow in a 5% CO_2_ atmosphere in the presence of H_2_O_2_, methyl viologen (MV, a quaternary ammonium compound that generates superoxide radicals) and diamide (a specific oxidant of thiols). In the presence of 0.2 mM H_2_O_2_, the Δ*suf* strain showed an extended lag phase and slow growth rates whereas the Δ*dpr* strain was completely unable to grow under this condition. In the presence of diamide, growth rates and growth yields of the Δ*suf* were severely affected (doubling time 3.54 ± 0.41 h compared to 1.48 ± 0.08 for the parent strain). The Δ*suf* strain also displayed long lag and slow growth rates in MV. Surprisingly, the Δ*dpr* strain that failed to grow in H_2_O_2_ was able to grow in the presence of diamide or MV, albeit with extended lag phases. Growth kinetics of the remaining strains, Δ*smu143c*, Δ*smu144c*, Δ*feo*, Δ*smu929*, Δ*smu1296* and Δ*smu1645*, in the presence of the different oxidative stress agents was nearly identical to that of the parent strain.

**Fig 4:**
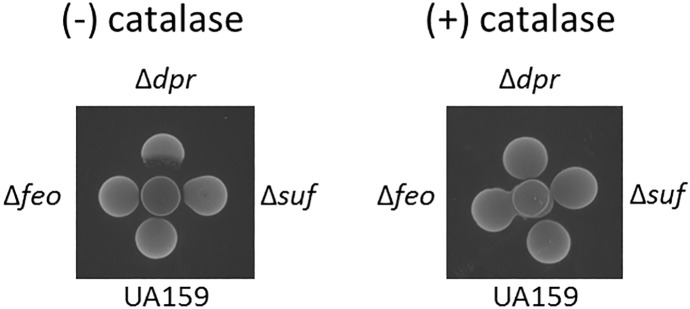
Growth competition on solid media between the peroxigenic *S*. *gordonii* and *S*. *mutans* UA159 and its derivatives. Competition assay reveals that *S*. *mutans* Δ*dpr* is sensitive to the H_**2**_O_**2**_ produced by *S*. *gordonii* (center spot). Under the conditions tested, growth of UA159, Δ*feo* and Δ*suf* strains (shown) as well as Δ*smu143*, Δ*smu144*, Δ*smu929*, Δ*smu1296* and Δ*smu1645* (not shown) was not inhibited by *S*. *gordonii*. The assay was repeated with catalase overlaid onto the *S*. *gordonii* spot to inactivate the H_**2**_O_**2**_, resulting in complete loss of sensitivity for the Δ*dpr* strain.

We also tested the ability of our panel of mutants to grow in the presence of the peroxigenic commensal *Streptococcus gordonii*. In agreement with its inability to grow in the presence of H_2_O_2_ (Fig 3), growth the Δ*dpr* strain was inhibited by *S*. *gordonii* (Fig 4). Despite an extended lag phase in media containing H_2_O_2_, growth of the Δ*suf* strain was not inhibited by *S*. *gordonii*. Also in agreement with growth curve results, the remaining mutant strains were not inhibited by *S*. *gordonii*, at least under the conditions tested (Fig 4 or data not shown).

### Iron sensitivity of the mutant strains

**Fig 5:**
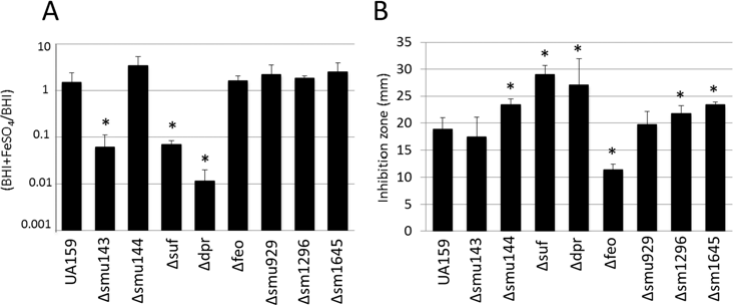
Iron homeostasis in *S*. *mutans* UA159 and its derivatives. (A) Growth of *S*. *mutans* UA159 and single mutant strains in the presence of 5 mM FeSO_**4**_. (B) Inhibition zones (in mm) of cultures in the presence of filter disks soaked with streptonigrin. (*) *p* ≤ 0.05.

Functional annotation of genes selected for this study suggest that some of the genes may be involved in metal ion homeostasis, which includes genes encoding a polypeptide deformylase metalloprotein (*smu143c*), an Fe-S cluster assembly system (*smu247-smu251*), iron-binding protein (*dpr*), iron transport system (*feoABC*) and tellurite resistance protein (*smu1645*). Metal ions, in particular iron, are essential for life functions, but cytoplasmic levels must be tightly controlled as iron can serve as a catalyst for the production of damaging hydroxyl radicals in the presence of H_2_O_2_ via Fenton reaction. Next, we tested the ability of our panel of mutant strains to cope with iron by assessing their ability to grow in the presence of 5 mM FeSO_4_ or streptonigrin, an iron-activated antibiotic [34] (Fig 5). Strains *Δsmu143*, *Δsuf* and Δ*dpr* displayed a growth impairment in the presence of FeSO_4_, whereas *Δsmu144*, *Δsuf*, Δ*dpr*, *Δsmu1296* and *Δsmu1645* showed increased sensitivity to streptonigrin based on disc diffusion assays. In agreement with its assigned role in iron transport, the *Δfeo* strain showed increased tolerance to streptonigrin but not to FeSO_4_. Because *smu1645* encodes a putative tellurite resistance protein, we also compared the minimum inhibitory concentration (MIC) of tellurite between the parent UA159 and *Δsmu1645* strains. In accordance with its predicted function, the Δ*smu1645* strain had a lower MIC for tellurite than UA159 (3.9 μM versus 15.6 μM for UA159, data not shown).

### The Δ*suf* and Δ*dpr* strains showed an impaired ability to colonize the teeth of rats

**Fig 6:**
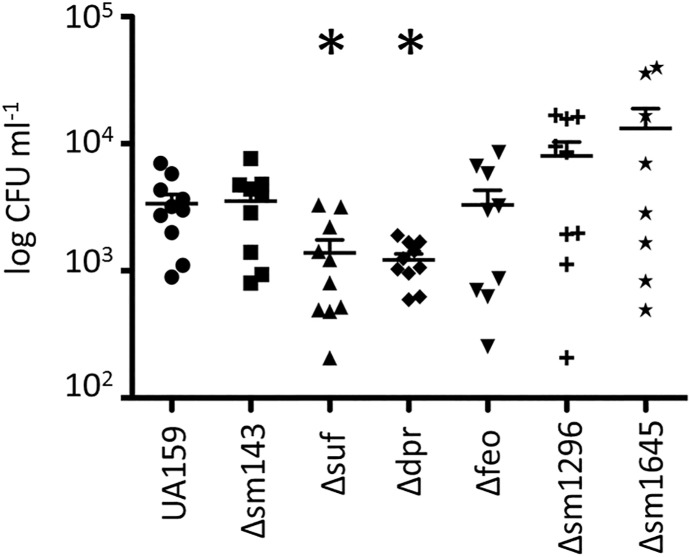
Colonization of *S*. *mutans* UA159 and derivative strains on the teeth of rats 15 days post-infection. The symbols represent the recovered bacterial colonies from each individual rat while the horizontal line represents the mean recovery per bacterial strain. (*) *p* ≤ 0.05.

**Fig 7:**
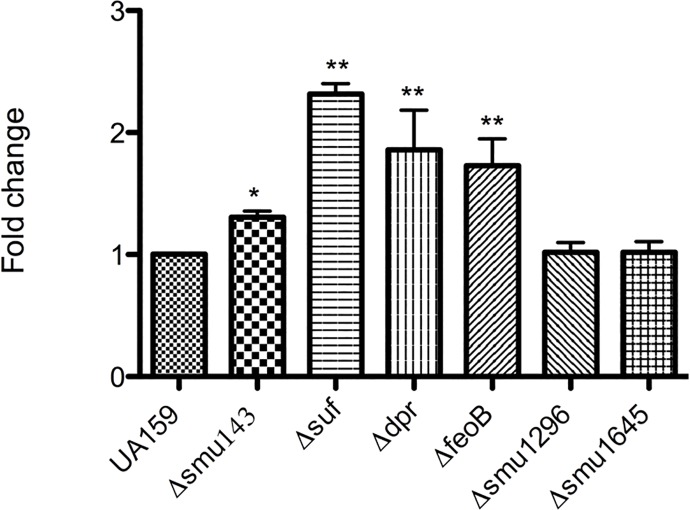
Biofilm formation by *S*. *mutans* UA159 and its derivatives. Cultures were gown for 24-h in BM supplemented with sucrose on the surface of 96-well microtiter plates. The graph shows the average and standard deviation for at least three independent experiments. (*) *p* ≤ 0.05.

Next, we tested the ability of the Δ*smu143*, Δ*suf*, Δ*dpr*, Δfeo, Δ*smu1296* and Δ*smu1645* strains to colonize the teeth of Wistar rats (Fig 6). Following a 15-day infection period, colonies of *S*. *mutans* were recovered from the jaws of infected animals by plating the jaw sonicates on MS agar. In agreement with the growth defect and general stress sensitivity, the infectivity of the Δ*dpr* and Δ*suf* strains was significantly reduced (*P* < 0.05). The differences observed between the remaining mutant strains were not statistically significant. Because the infectivity of *S*. *mutans* in the oral cavity has been shown to be directly tied to its ability to form biofilms on tooth surfaces through a sucrose-dependent mechanism [35], we also evaluated the capacity of each strain to form biofilms on microtiter plates in the presence of sucrose. The Δ*smu143*, Δ*dpr* and Δ*suf* and Δ*feoB* strains accumulated more biofilm (*P* < 0.05) when grown in sucrose when compared to UA159 (Fig 7). Therefore, the lower infectivity of the Δ*dpr* and Δ*suf* strains in the teeth of rats does not appear to correlate with a deficiency in biofilm formation.

### A phenotype enhancement screen confirmed the physiologic relevance of the Fe-S cluster assembly system but failed to assign new roles to the other Spx-regulated genes

**Fig 8:**
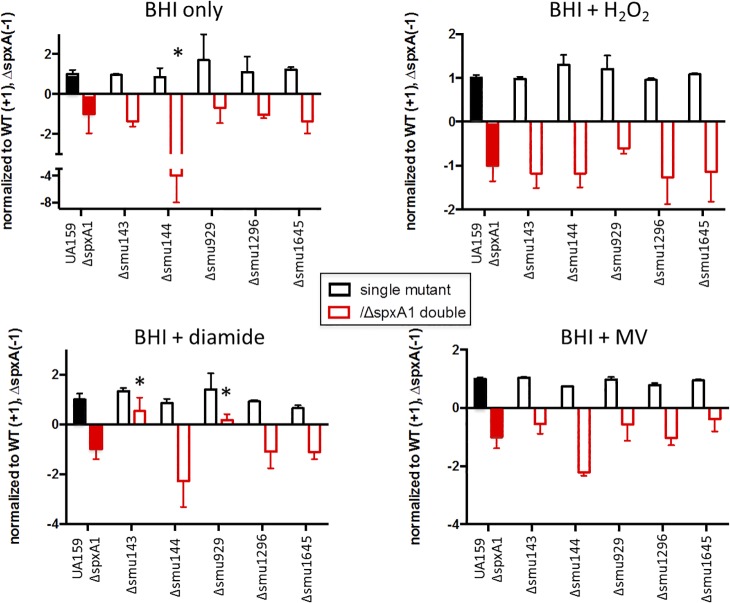
Phenotype enhancement screen of Spx-regulated genes. Growth of single mutants (Δ*smu143*, Δ*smu144*, Δ*smu929*, Δ*smu1296* and Δ*smu1645*, open black bars) and double (Δ*smu/*Δ*spxA1*, open red bar) in relation to wild-type UA159 and Δ*spxA1* single mutant. Solid bars represent normalized growth of Δ*spxA1* strains (grey bar, set to -1) in relation to UA159 (black bar, set to 1). (*) *p* ≤ 0.05.

Due to functional redundancy and genetic buffering, it is not unusual that single gene deletions do not have any detectable phenotype. This is readily observed in systematic gene deletion libraries [36,37] whereby gene functions are rarely assigned based on single deletions. A useful way to overcome this limitation is the construction of paired mutations that may eliminate or reduce genomic buffering effects [38,39]. Recently, the Zuber lab utilized such an approach to uncover the role of Spx-regulated genes of unknown function in *B*. *subtilis* [40]. Specifically, double mutant strains were created by introducing mutations on selected Spx-regulated genes into the *spx* mutant background followed by a screen for enhanced oxidative stress sensitivity when compared to the *spx* single mutant. Here, we utilized a similar approach by inactivating our genes of interest, one at a time, in the Δ*spxA1* strain. We have previously shown that the Δ*spxA1* strain grew poorly under oxidative stress conditions [19], thus we evaluated the ability of Δ*spxA1* single and double mutants to grow under these conditions. While we were able to isolate a Δ*spxA1*Δ*suf* double deletion strain, this strain grew very poorly under standard laboratory conditions and was not amenable to further phenotypic characterization. In most cases, the double mutants did not display slower growth rates or hypersensitivity against the oxidative stress agents tested (H_2_O_2_, MV or diamide) when compared to the Δ*spxA1* single mutant (Fig 8). The only exceptions were the Δ*spxA1*Δ*smu143c* and Δ*spxA1*Δ*smu929c* strains that, unexpectedly, grew better than Δ*spxA1* in the presence of diamide and the Δ*spxA1*Δ*smu144c* strain that showed slower growth than Δ*spxA1* in the presence of MV. We tested whether the increased diamide resistance of the Δ*spxA1*Δ*smu143c* and Δ*spxA1*Δ*smu929c* strains was a result of elevated *spxA2* expression but in, both case, the transcriptional levels of *spxA2* were identical to the parent UA159 strain (S5 Fig). At this time, the reasons for mutations on either the *smu143* or the *smu929* genes serving to alleviate the diamide sensitivity of the Δ*spxA1* strain are unknown. One possible explanation is that loss of Smu143 or Smu929 triggered some yet to be determined oxidative stress mechanism that compensates for the loss of SpxA1.

## Discussion

While the significance of acid production, acid tolerance and biofilm formation to the cariogenic potential of *S*. *mutans* has been well established and have been studied in great detail [2], the role of bacterial oxygen metabolism and oxidative stress survival in caries have thus far received limited attention. Over the years, the primary notion that the dental plaque environment was virtually anaerobic has been replaced by evidence that, as a whole, the microbial plaque community has a high capacity to reduce oxygen thereby generating a variety of toxic byproducts. The relevance of oxidative stress survival to the pathophysiology of *S*. *mutans* has been further supported by *in vitro*, *in vivo* and clinical studies, showing an inverse relationship between the presence of H_2_O_2_-generating streptococci from the mitis group and *S*. *mutans* [5,41–43].

Spx proteins are conserved global transcriptional regulators of Gram-positive bacteria that function as positive regulators of oxidative stress genes [15] that, more recently, have been linked to the virulence potential of *E*. *faecalis*, *S*. *mutans* and other streptococcal species [19,22,23,44]. Previously, using microarrays, we showed that most genes with a proven role in the oxidative stress response of *S*. *mutans* were under SpxA1 positive regulation, and, to a lesser extent, SpxA2 [19]. Specifically, expression of genes involved in oxygen metabolism (*nox*), thiol stress (*trxA* and *trxB*) and ROS detoxification (*ahpC*, *ahpF*, *sodA* and *tpx*) among others was significantly lower in strains lacking *spxA1*, *spxA2* or both. Here, we sought to uncover the function of previously uncharacterized genes that, in the microarrays, displayed the same regulatory profile of the known oxidative stress genes, namely *smu127*, *smu143*, *smu144*, *smu247*, *smu929c*, *smu1296*, *smu1297*, and *smu1645*. In addition, we included one previously characterized oxidative stress gene, *smu540* (*dpr*), to serve as a phenotypic benchmark as well as to gain additional knowledge about the function of Dpr in *S*. *mutans*. An exception to the trend of positive regulation by Spx was the inclusion of the *smu569/570/571* transcriptional unit encoding the FeoABC iron transport system, which was by our microarrays to be negatively regulated by SpxA1. Given the well-defined role of iron in hydroxyl radical formation as a catalyst of the Fenton reaction, it is therefore expected that negative regulation of iron uptake by Spx is an integral part of the oxidative stress response.

The transcriptional data obtained from our microarrays provided insight into the role of Spx proteins as regulators under basal conditions, when the cells were not subjected to oxidative stress. Here, quantitative real time PCR analysis of cells exposed to H_2_O_2_ further supported that the genes studied herein are regulated by Spx and encode bona fide oxidative stress proteins. Exposure to H_2_O_2_ resulted in increased expression of each of these genes (with the exception of, as expected, *feoB*), demonstrating a role for each of them in defending the cell against oxidative stresses. However, in the absence of SpxA1, exposure to oxidative stress did not trigger increased expression of these genes, demonstrating their regulatory dependence upon SpxA1. With the exception of *smu143c* (polypeptide deformylase), *smu144c*, (transcriptional regulator) and *smu929c*, which encodes a hypothetical membrane protein conserved among Gram-positive cocci, all other genes are predicted to encode proteins involved in oxidative stress responses in other bacteria. Among the latter group of genes, the aforementioned *dpr* and *feoB* are predicted to participate in oxidative stress defenses by binding to iron (Dpr) or by limiting iron uptake (FeoB). The FeoABC system can be found in both Gram-positive and Gram-negative bacteria and has been implicated in virulence of several Gram-negative species as well as *Streptococcus suis* [45,46]. With the exception of the increased resistance to streptonigrin, an antibiotic whose toxicity depends on the availability of intracellular free iron, the Δ*feo* strain phenocopied the parent strain including a nearly identical ability to colonize the teeth of rats fed a cariogenic diet. This may be explained, at least in part, by iron being non-specifically transported by other metal-binding ABC transporters. The iron-binding Dpr has been extensively studied in bacteria and shown to mitigate H_2_O_2_ lethality by preventing the Fenton reaction [11,33]. In agreement with previous reports [11,14,47], the Δ*dpr* strain was hypersensitive to H_2_O_2_ and, based on indirect evidence, including growth in the presence of iron and tolerance to streptonigrin, cannot maintain iron homeostasis. Interestingly, growth of the Δ*dpr* strain was not affected by diamide, and only modestly inhibited by MV. However, the different behavior of the Δ*dpr* mutant in the presence of H_2_O_2_, diamide or MV is on par with a proteomic study in *S*. *aureus* that revealed a small overlap in the kinds of proteins produced when cells are exposed to these oxidants [48]. Diamide specifically oxidizes thiol groups, causing an increase in formation of intra- or extramolecular disulfide bonds leading to the accumulation of misfolded and aggregated proteins as a result of thiol oxidation. In this case, hydroxyl radical formation due to Fenton reaction should play an indirect role in cell viability. MV, also known as paraquat, generates O_2_^-^ radicals in the cell that can directly mobilize Fe-S clusters or can be converted to H_2_O_2_. It is conceivable that the Δ*dpr* strain can partially overcome the detrimental effects of MV by protecting Fe-S cluster enzymes, or by activating multidrug efflux systems. In fact, an ABC transporter, dubbed VltA/VltB for viologen transporter, was shown to mediate resistance to MV as well as other quaternary ammonium compounds in *S*. *mutans* [49]. Most importantly, the Δ*dpr* mutant showed reduced ability to colonize the teeth of rats thereby providing, for the first time, direct evidence that Dpr is involved in the virulence of *S*. *mutans*.

The most dramatic phenotypes were observed in the Δ*suf* strain, in which the entire *suf* operon was inactivated with a polar kanamycin cassette. The Δ*suf* strain displayed impaired growth under all conditions tested, increased sensitivity to iron and lower infectivity in rats. The SUF machinery is one of three Fe-S cluster assembly systems identified in bacteria but can also be found in plant chloroplasts [50]. Proteins containing Fe-S clusters as a prosthetic group are widely distributed in nature and perform essential biological processes including electron transfer, substrate binding/activation, transcriptional and translational regulation and iron storage [51]. ROS cause destabilization of Fe-S clusters affecting important cellular processes and stimulating the Fenton reaction due to an increase in intracellular free iron. Two Fe-S assembly systems are present in *E*. *coli*: the housekeeping ISC and the stress-inducible SUF, whereas only the SUF machinery is present in *S*. *mutans* and other members of the Firmicutes phylum [52]. Considering that the SUF system is the only pathway for assembly and repair of Fe-S clusters in *S*. *mutans*, it was not entirely unexpected that fitness and viability of the Δ*suf* strain was significantly impaired. Interestingly, while transcriptionally induced by H_2_O_2_ stress and dependent on the SpxA1 and SpxA2 regulators for optimal expression (see Fig 1), our IVT assays indicate that neither SpxA1 nor SpxA2 directly induce transcription of the *suf* operon. The most logical interpretation of these results is that Spx exerts indirect control over *suf* gene expression perhaps by controlling the transcription of another regulator. Of note, the *suf* operon of *E*. *coli* is induced by oxidative stress and is under the control of three transcriptional regulators, IscR, Fur and OxyR [53,54].

Although strains lacking either *smu144c* or *smu143c* were able to grow as well as the parental strain under oxidative stress conditions, it is important to note that the *smu144c*/*143c* genes, which are likely co-transcribed, were: (i) induced by H_2_O_2_ treatment, (ii) directly regulated by SpxA1, and apparently (iii) unable to maintain iron homeostasis. The *smu144c* genes encode a putative transcriptional regulator of the Crp/Fnr family conserved in many streptococcal species. Members of the Crp/Fnr family utilize Fe-S clusters to sense and respond to oxygen. The *smu43c* codes for a polypeptide deformylase (PDF) metalloenzyme, which removes the formyl group from the N-terminal methionine of newly synthesized proteins. The crystal structure of Smu143c has been solved (pdb3L87) and shown to coordinate binding to ferrous ion. Given that the PDF process is essential in bacteria but not in eukaryotes, PDF inhibitors are considered promising targets for the development of new antimicrobials [55].

In conclusion, we have shown that the Spx proteins of *S*. *mutans* control the expression of several additional genes involved in oxidative stress management. It appears that in addition to the transcriptional activation of oxygen metabolism, ROS scavengers and thiol repair enzymes, Spx performs an important role in iron homeostasis by regulating the intracellular availability of free iron. As part of a continued effort to assign a functional role for the genes characterized in this study, an expanded phenotype enhancement screen based on the double *spx* strain (Δ*spxA1*Δ*spxA2*) is currently under way.

## Supporting Information

S1 Table Real-time PCR primers. (DOCX)

S2 Table Primers used for gene inactivation. (DOCX)

S3 Table *In vitro* transcription primers. (DOCX)

S1 Fig Transcriptional shift profiles derived from RT-qPCR after 5 and 15 minute exposures to % H_2_O_2_, presented as the log base 2 average fold change from pre-treatment transcript copy numbers of at least three replicates. Heat map construction was performed in R (http://www.R-project.org/) version 2.1.0, using packages 'gplots' (2.14.1) and 'RColorBrewer' (1.0–5).
(PPT)

S2 Fig Schematic representation of the *smu143c/144c*, *sufCDSUB*, *dpr*, *feoABC*, *smu929c*, *smu1296* and tehB loci in *S*. *mutans* UA159. (PPT)

S3 Fig RT-PCR analysis of the *smu144c*-*smu143c* transcriptional unit. Products from the PCR were derived from *S*. *mutans* UA159 chromosomal DNA (positive control, lane 1), cDNA obtained from total mRNA (lane 2) and a negative control using total mRNA but omitting RT (negative control, lane 3).
(PPT)

S4 Fig RT-qPCR analysis of (A) *smu143c* gene expression in the Δ*smu144* strain and (B) *smu1297* expression in the Δ*smu1296* strain. Total RNA was isolated from mid-exponential phase cultures grown in BHI at 37°C. Bars represent the relative copy number detected for each gene.
(PPT)

S5 Fig RT-qPCR analysis of *spxA2* gene expression in the Δ*smu143*/Δ*spxA1* and Δ*smu929/*Δ*spxA1* strains. Total RNA was isolated from mid-exponential phase cultures grown in BHI at 37°C. Bars represent the relative copy number detected for each gene.
(PPT)
