# Supplementary material for: Transcriptional and Phenotypic Characterization of Novel Spx-Regulated Genes in Streptococcus mutans
Source: PLoS One. 2015 Apr 23;10(4):e0124969. doi: 10.1371/journal.pone.0124969 (PMC4408037; doi:10.1371/journal.pone.0124969)
Supplement: S1 Table — (DOCX) [file pone.0124969.s001.docx]

**Table S1.** **Primers used for gene inactivation.**

| **Primer** | **Sequence** | **Application** |
| --- | --- | --- |
| 5'143Arm1 | 5'-GCAGTAAGACCGCTAATATAGC-3' | *smu143c deletion* |
| 3-143Arm1 | 5'-AATAGCAGGTACCTTTTTCTCC-3' |  |
| 5'143Arm2 | 5'-TTGATTGAAGCTTGGTACTTAGC-3' |  |
| 3'143Arm2 | 5'-CGTCAGTGACACGGC-3' |  |
| 5'144Arm1 | 5'-CCATAATGATTAGCCTGC-3' | *smu144c* deletion |
| 3'144Arm1KpnI | 5'-CCATTCTTTGGTACCAGCCACAAG-3' |  |
| 5'144Arm2KpnI | 5'-CGCTGGCAAGGTACCGAATAAGGC-3' |  |
| 3'144Arm2 | 5'-CCCTAGTTCTGTCCTTTGT-3' |  |
| 5'248Arm1 | 5'-GTTTAGAGATTTGGGGAC-3' | *smu247/248 deletion* |
| 3'248Arm1BamHI | 5'-GCTCTGCCTGGATCCGTGAAAATG-3' |  |
| 5'248Arm2BamHI | 5'-GTGACGAAAGGATCCCTGTTTTGG-3' |  |
| 3'248Arm2 | 5'-GGTCCATAGACTGTCAAC-3' |  |
| 5’540Arm1 | 5'-GCCAGCTAATGTCAGAAACAC-3' | *smu540 deletion* |
| 3’540Arm1HindIII | 5'-GCAGAATCAAGCTTTTTGACC-3' |  |
| 5’540Arm2HindIII | 5'-CGTGGTTAAAGCTTGACCTTG-3' |  |
| 3’540Arm2 | 5'-CGCGATTAGTCACAATTTTATC-3' |  |
| 5'570Arm1 | 5'-CGTACTTCTTTTTGATGAG-3' | *smu569/570* deletion |
| 3'570Arm1BamHI | 5'-CGCTATTGGGGATCCCAATCAAAG-3' |  |
| 5'570Arm2BamHI | 5'-CCACGTCAGGGATCCGAAAAAGTG-3' |  |
| 3'570Arm2 | 5'-CACCTTCTTTGACAAATTC-3' |  |
| 5'929Arm1 | 5'-CAGGTTCTTTTAGTGAAGCTGC-3' | *smu929c deletion* |
| 3'929Arm1KpnI | 5'-GCATTTTAATAATTGGTACCATTGCC-3' |  |
| 5'929Arm2HindIII | 5'-CGCTGCAAAGCTTTATCTCTT-3' |  |
| 3'929Arm2 | 5'-ACAGAAGAACAGGCAGAGTC-3' |  |
| 5'1296Arm1 | 5'-GGTTCCGAAATAATCCACA-3' | *smu1296* deletion |
| 3'1296Arm1KpnI | 5'-GACCAAACTTTGGTACCAATAAGTAAG-3' |  |
| 5'1296Arm2KpnI | 5'-GGCACAAAGGGTACCAGTTCTTAAC-3' |  |
| 3'1296Arm2 | 5'-GGGAAAGAATCCATTTGACGT-3' |  |
| 5'1645Arm1 | 5'-GACCAGGACATTTGTATAG-3' | *smu1645* deletion |
| 3'1645Arm1KpnI | 5'-GCCAGCAGCATAGGTACCTTTCTGTTAG-3' |  |
| 5'1645Arm2KpnI | 5'-CCTTGGATCGGTACCAGGACGTAA-3' |  |
| 3'1645Arm2 | 5'-GCTCTAAAATGCATCGTC-3' |  |
