# Supplementary material for: Transcriptional and Phenotypic Characterization of Novel Spx-Regulated Genes in Streptococcus mutans
Source: PLoS One. 2015 Apr 23;10(4):e0124969. doi: 10.1371/journal.pone.0124969 (PMC4408037; doi:10.1371/journal.pone.0124969)
Supplement: S2 Table — (DOCX) [file pone.0124969.s002.docx]

**Table S2**. **Real-time PCR primers.**

| **Gene ID** | **Primer** | **Product size (bp)** | **Sequence** |
| --- | --- | --- | --- |
| *smu127* | 5'127Arm1 | 150 | 5’-CGTGCTGATGCTTATGGTATTC-3’ |
|  | 3'127Amr2 |  | 5’-TCCGAACCAACGATAAGATTCC-3’ |
| *smu143c* | 5'143Arm1 | 178 | 5'-TTGCTGTTCTCATTCCTAAT-3' |
|  | 3'143Arm2 |  | 5'-CGGATAACATAGCCTTCAA-3' |
| *smu144c* | 5'144Arm1 | 168 | 5'-TTATTGGAGATATTGAACTGCTAA-3' |
|  | 3'144Arm2 |  | 5'-TGGTAGAGTGAATACAA-3' |
| *smu248* | 5'248Arm1 | 193 | 5'-ACCAACTACGGCAATAAC-3' |
|  | 3'248Arm2 |  | 5'-CATCAATAAGAAGAATAGGATTAGC-3' |
| *smu540* | 5'540Arm1 | 162 | 5'-GAAGAAACAGTTGGCACATGGG-3' |
|  | 3'540Arm2 |  | 5'-TTCCGTTTGAGCTGCTGTAAAG-3' |
| *smu570* | 5'feoBArm1 | 131 | 5'-TTTAACAGGGACAAGTCAG-3' |
|  | 3'feoBArm2 |  | 5'-ATTATACCATTGCCGATA-3' |
| *smu929c* | 5'929Arm1 | 123 | 5'-TCTACAAAGACATCTCAAGTT-3' |
|  | 3'929Arm2 |  | 5'-GCCATAAAGCAGACCTAA-3' |
| *smu1296* | 5'1296Arm1 | 116 | 5'-ATTATACCATTGCCGATA-3' |
|  | 3'1296Arm2 |  | 5'-GTCTAATAAGTGTTGATAGG-3' |
| *smu1297* | 5'1297Arm1 | 119 | 5'-GGGATTCTTGGTGATACAG-3' |
|  | 3'1297Arm2 |  | 5'-TCCATTTGACGTGCTAAG-3' |
| *smu1645* | 5'1645Arm1 | 107 | 5'-TAGACCAGTTGTACCAGATA-3' |
|  | 3'1645Arm2 |  | 5'-TGGGACAAGGATAGGATT-3' |
