# Supplementary material for: Transcriptional and Phenotypic Characterization of Novel Spx-Regulated Genes in Streptococcus mutans
Source: PLoS One. 2015 Apr 23;10(4):e0124969. doi: 10.1371/journal.pone.0124969 (PMC4408037; doi:10.1371/journal.pone.0124969)
Supplement: S3 Table — (DOCX) [file pone.0124969.s003.docx]

**Table S3. *In vitro* transcription primers.**

| **Gene ID** | **Transcript size (bp)** | **Primer name** | **Sequence** |
| --- | --- | --- | --- |
| *smu127* | 80 | 5'127Arm1IVT | GACTGTTATAGTGGGATTCTTATCTG |
|  |  | 3'127Arm2IVT | CGCTGCATTTTTAAAAACATATCC |
| *smu144c* | 322 | 5'144Arm1IVT | CTCCTTAAACACAAAACTTG |
|  |  | 3'144Arm2IVT | CGGCAATTGGACAACCC |
| *smu247* | 118 | 5'247Arm1IVT | CAAGAAGAAGTCCAATTG |
|  |  | 3'247Arm2IVT | GCATGGCAAGAAAAAGCC |
| *smu540* | 70 | 5'540Arm1IVT | GCCAAGAGAAGCTCCCAAC |
|  |  | 3'540Arm2IVT | CTGAATTTTCTTTTTCTCCACTTGATG |
| *smu569* | 184 | 5'569Arm1IVT | CGGTGAGTACACTCAGC |
|  |  | 3'569Arm2IVT | GAATCATCAAAAGCTAAACG |
| *smu929c* | 144 | 5'929Arm1IVT | GGCAGAAAAAGTACAAAAATGTTCAC |
|  |  | 3'929Arm2IVT | GTGGCTAACGTTTCCAAATAC |
| *smu1296* | 96 | 5'1296Arm1IVT | CACATTTCAGTTGATTTTCTC |
|  |  | 3'1296Arm2IVT | GTCAAAACGACTGCCAG |
| *smu1645* | 72 | 5'1645Arm1IVT | CCACTCGTTTTGATTCATG |
|  |  | 3'1645Arm2IVT | GATGCTATTGGCAGTCC |
