# Supplementary figures and images for: Transcriptional and Phenotypic Characterization of Novel Spx-Regulated Genes in Streptococcus mutans
Source: PLoS One. 2015 Apr 23;10(4):e0124969. doi: 10.1371/journal.pone.0124969 (PMC4408037; doi:10.1371/journal.pone.0124969)

## Slide 1
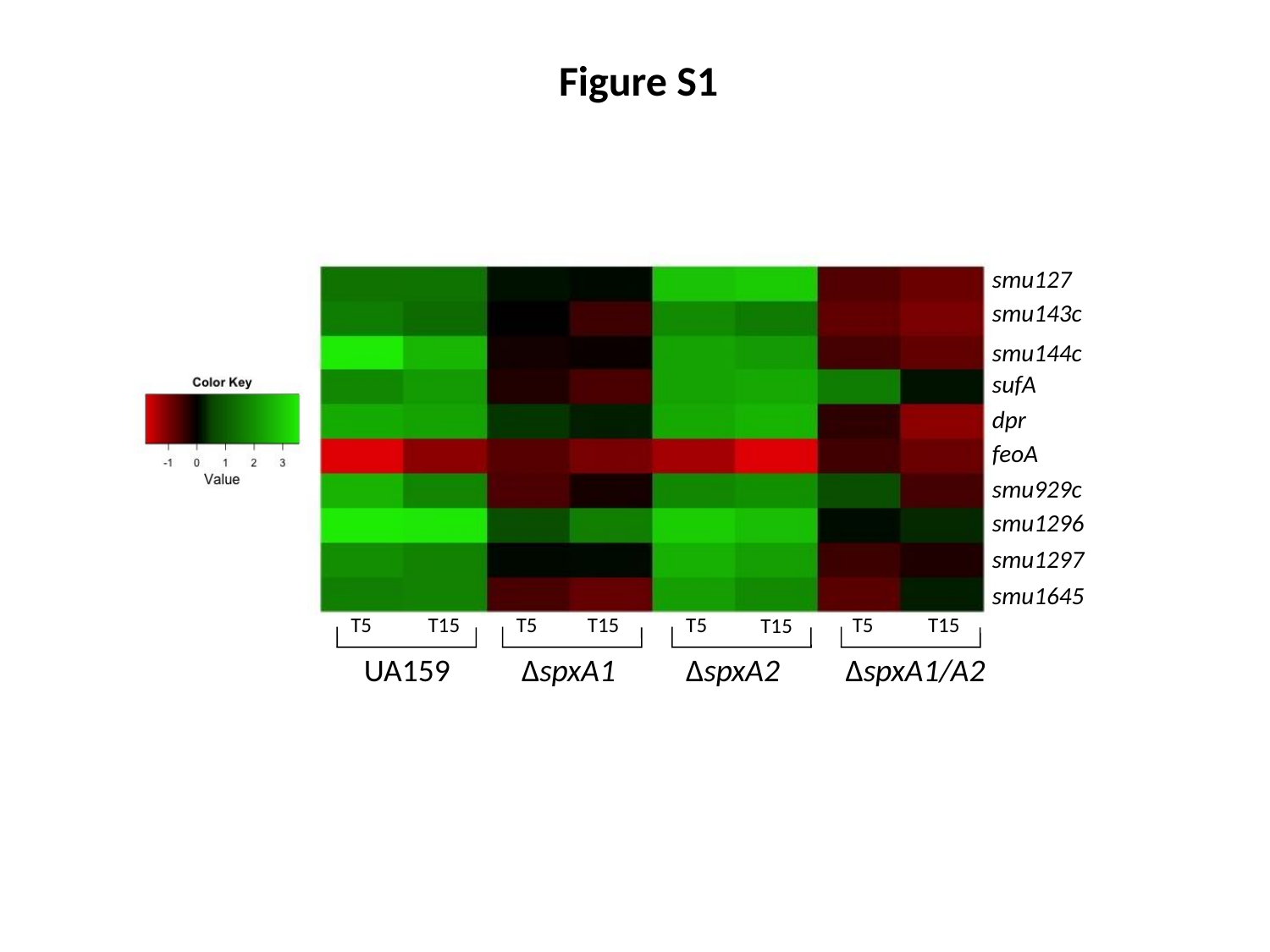

Figure S1
smu127
smu143c
smu144c
sufA
dpr
feoA
smu929c
smu1296
smu1297
smu1645
T5
T15
T5
T15
T5
T5
T15
T15
UA159
ΔspxA1
ΔspxA2
ΔspxA1/A2

Supplement: S1 Fig — Heat map construction was performed in R (http://www.R-project.org/) version 2.1.0, using packages 'gplots' (2.14.1) and 'RColorBrewer' (1.0–5). (PPT) [file pone.0124969.s004.ppt]

## Slide 1
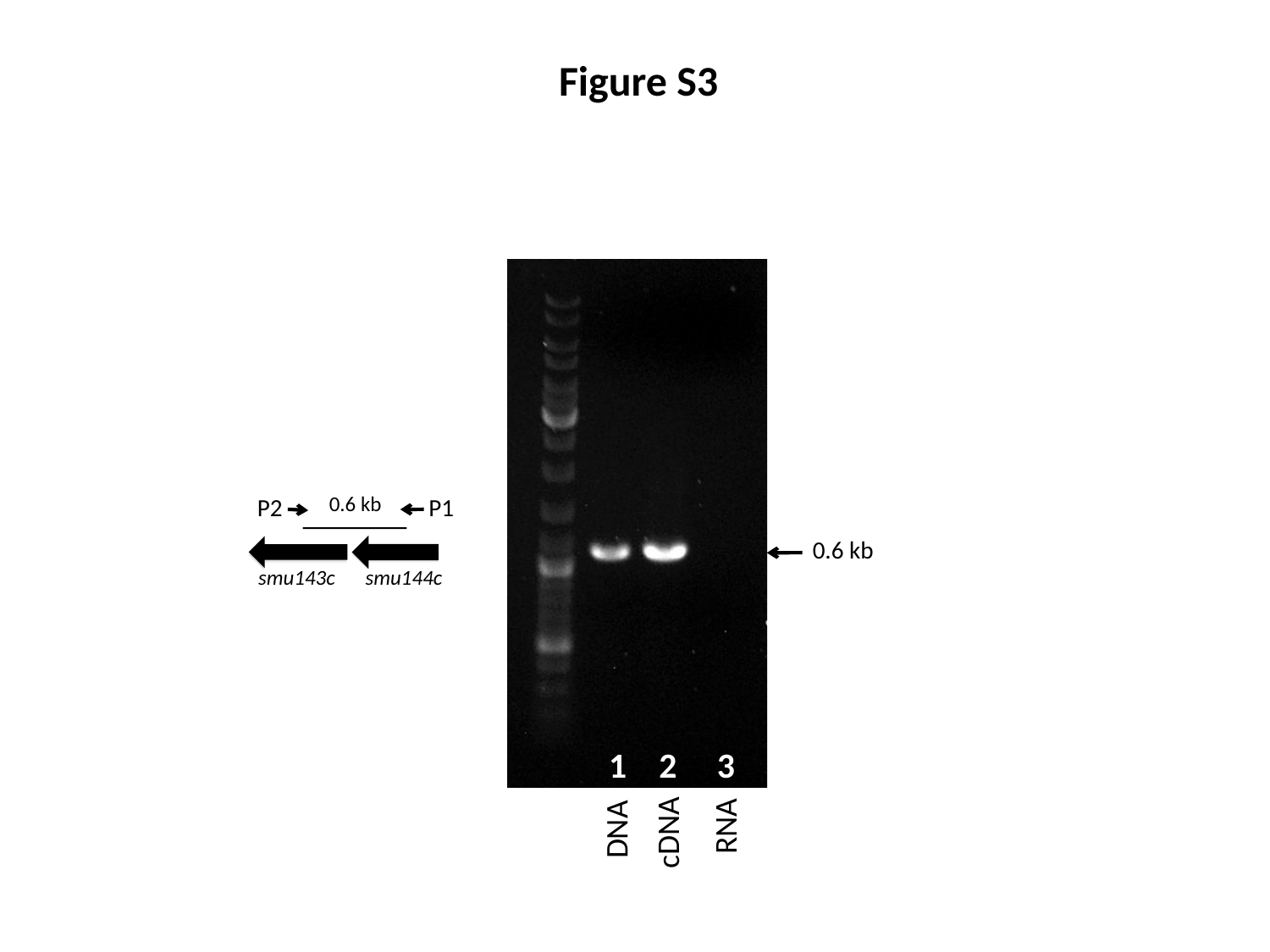

Figure S3
 1 2 3
RNA
DNA
cDNA
0.6 kb
P2
P1
smu143c
smu144c
0.6 kb

Supplement: S3 Fig — Products from the PCR were derived from S. mutans UA159 chromosomal DNA (positive control, lane 1), cDNA obtained from total mRNA (lane 2) and a negative control using total mRNA but omitting RT (negative control, lane 3). (PPT) [file pone.0124969.s006.ppt]

## Slide 1
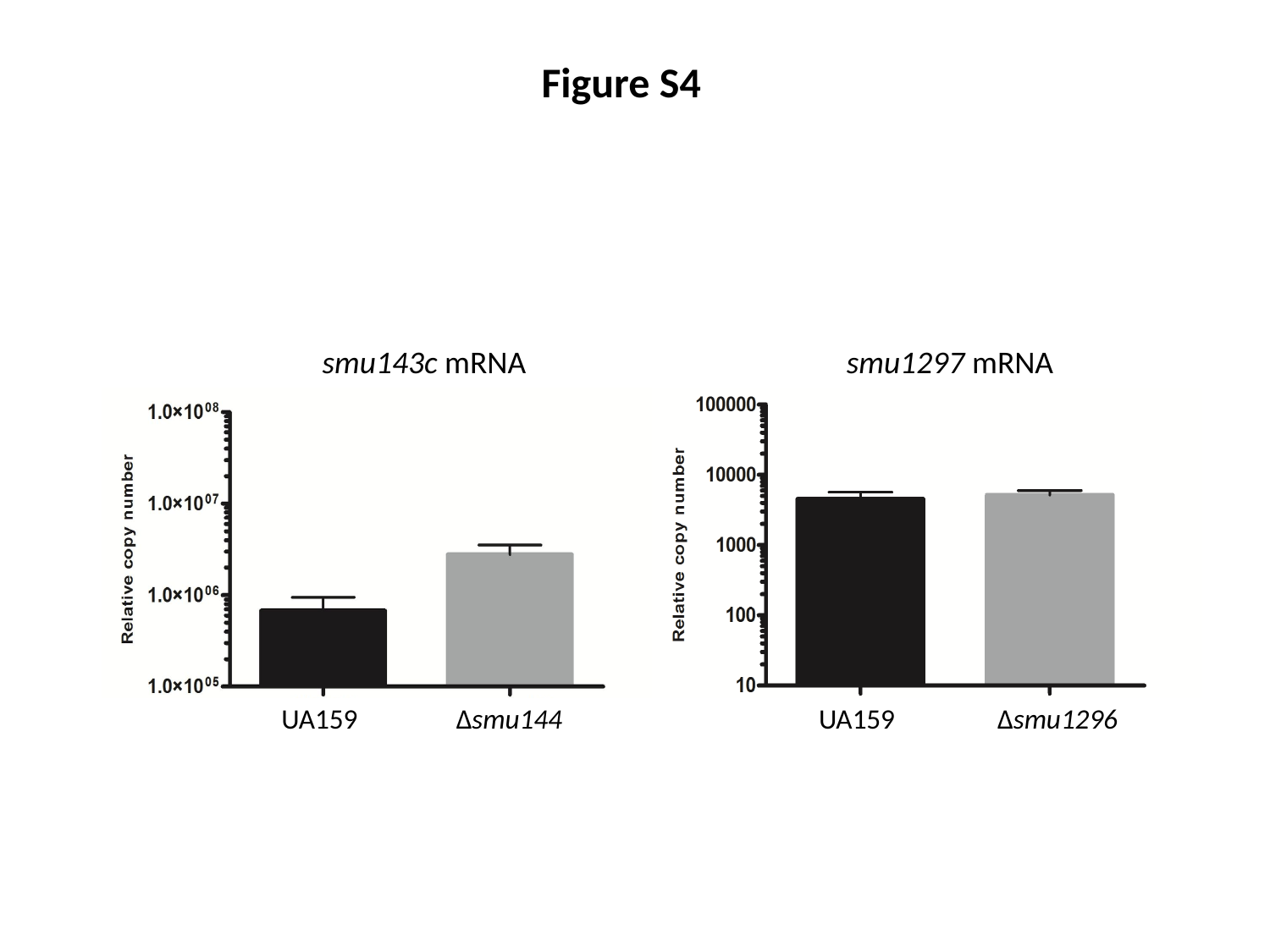

Figure S4
smu143c mRNA
smu1297 mRNA
UA159
Δsmu144
UA159
Δsmu1296

Supplement: S4 Fig — Total RNA was isolated from mid-exponential phase cultures grown in BHI at 37°C. Bars represent the relative copy number detected for each gene. (PPT) [file pone.0124969.s007.ppt]

## Slide 1
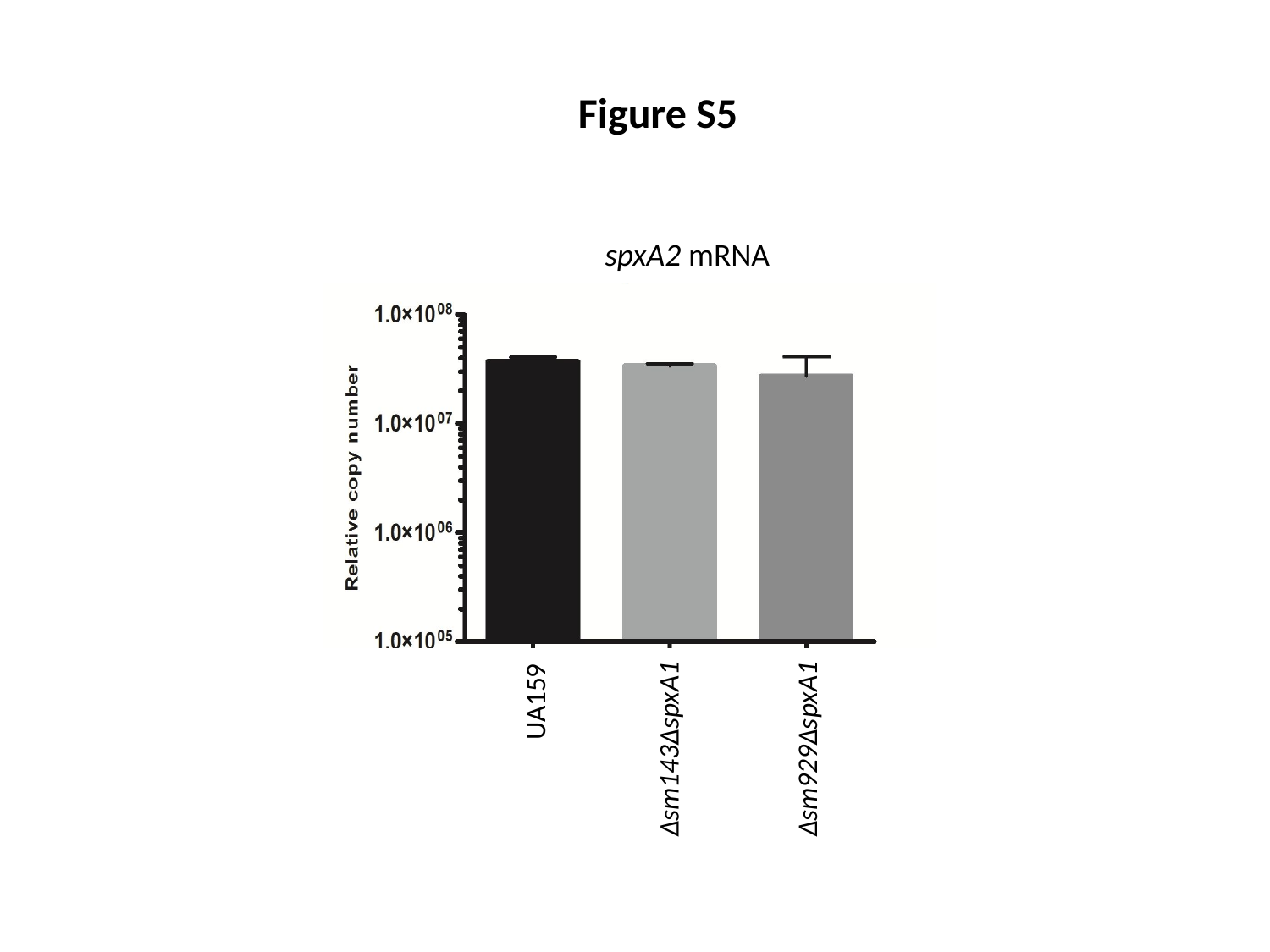

Figure S5
spxA2 mRNA
UA159
Δsm143ΔspxA1
Δsm929ΔspxA1

Supplement: S5 Fig — Total RNA was isolated from mid-exponential phase cultures grown in BHI at 37°C. Bars represent the relative copy number detected for each gene. (PPT) [file pone.0124969.s008.ppt]
