# Supplementary material for: Transcriptional and Phenotypic Characterization of Novel Spx-Regulated Genes in Streptococcus mutans
Source: PLoS One. 2015 Apr 23;10(4):e0124969. doi: 10.1371/journal.pone.0124969 (PMC4408037; doi:10.1371/journal.pone.0124969)
Supplement: S2 Fig — (PPT) [file pone.0124969.s005.ppt]

## Slide 1
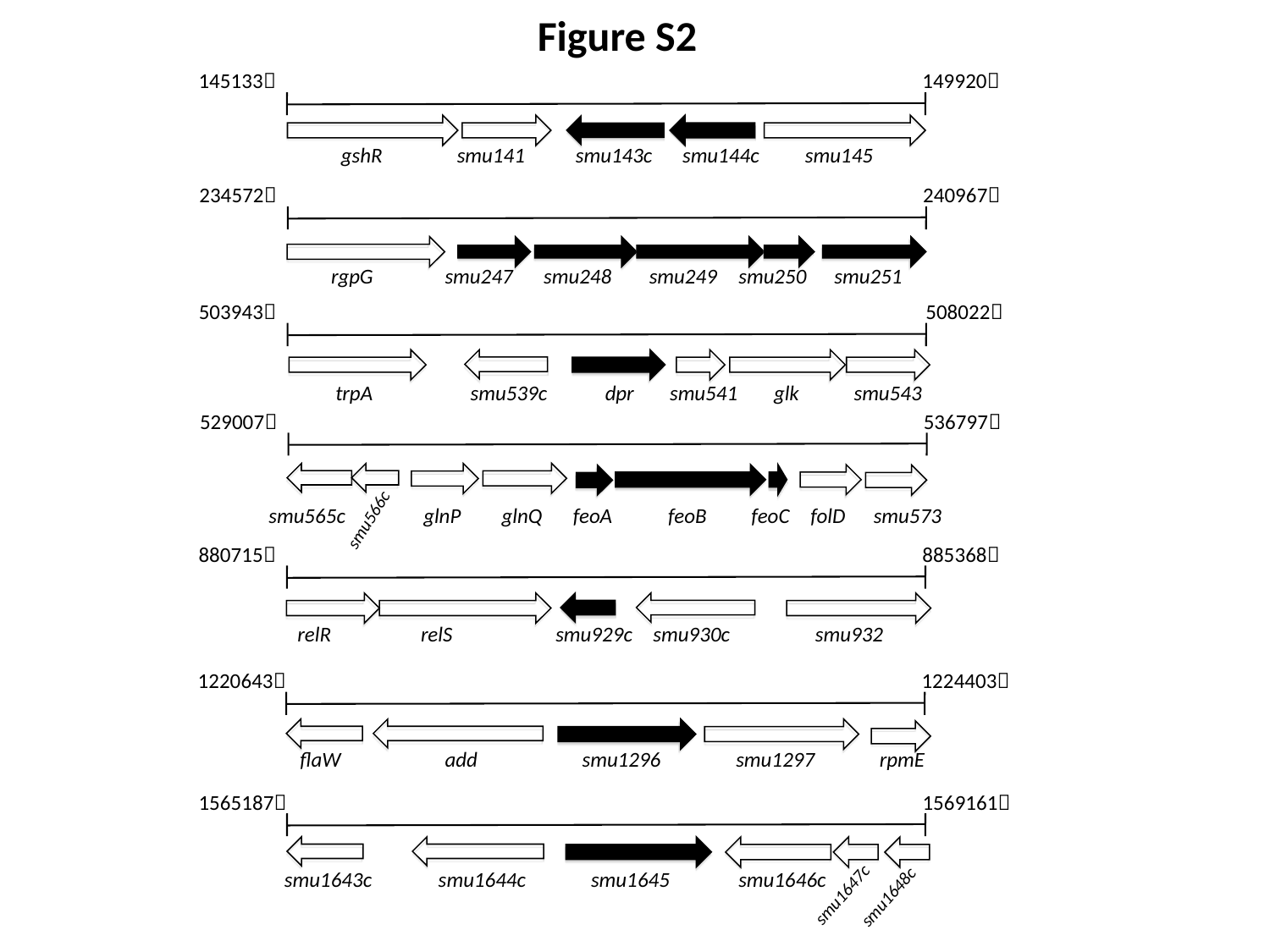

Figure S2
145133
149920
gshR
smu141
smu143c
smu144c
smu145
234572
240967
rgpG
smu247
smu248
smu249
smu250
smu251
503943
508022
trpA
smu539c
dpr
smu541
glk
smu543
529007
536797
smu565c
glnP
glnQ
feoA
feoB
folD
smu573
smu566c
feoC
880715
885368
relR
relS
smu929c
smu930c
smu932
1220643
1224403
flaW
add
smu1296
smu1297
rpmE
1565187
1569161
smu1643c
smu1644c
smu1645
smu1646c
smu1647c
smu1648c
